# Genetic Variability of Loci Affecting Meat Quality and Production in Nero Siciliano Pig Breed

**DOI:** 10.3390/ani15142143

**Published:** 2025-07-19

**Authors:** Serena Tumino, Morena Carlentini, Giorgio Chessari, Andrea Criscione, Aurora Antoci, Donata Marletta, Salvatore Bordonaro

**Affiliations:** Dipartimento di Agricoltura, Alimentazione e Ambiente, University of Catania, 95123 Catania, Italy; serena.tumino@unict.it (S.T.); morena.carlentini@phd.unict.it (M.C.); giorgio.chessari@unict.it (G.C.); andrea.criscione@unict.it (A.C.); aurora.antoci@studium.unict.it (A.A.); s.bordonaro@unict.it (S.B.)

**Keywords:** Nero Siciliano pig breed, genetics, meat quality, biodiversity, safeguarding

## Abstract

Nero Siciliano (NS) is an autochthonous pig breed reared in northeastern Sicily; despite its high-quality meat products, NS is currently endangered. This study aimed to evaluate the genetic variability at nine loci within candidate genes for meat traits—Melanocortin 4 Receptor (*MC4R*), Ryanodine Receptor 1 (*RYR1*), Class 3 Phosphoinositide 3-Kinase (*PIK3C3*) and Leptin (*LEP*)—to provide useful information for preservation and exploitation of the NS pig breed. Distribution of the genetic variants was assessed in a representative sample of 87 pigs (18 boars and 69 sows) collected in nine farms located in the original breeding area. Genotypes have been determined using PCR-RFLP and Sanger sequencing. Alleles linked to different growth rates and back fat deposition showed high frequencies (*MC4R* c.175C—0.93; *LEP* g.3469T—0.91) in the whole sample. Deviations from Hardy–Weinberg equilibrium and different allele distribution in boars and sows were observed. The *RYR1* g.1843T allele, associated with Malignant Hyperthermia and Pale Soft Exudative meat defect, was reported in seven heterozygote pigs (q = 0.04) with one farm exhibiting a frequency of 0.29. Our results suggest the need for continuous monitoring of the genetic variants in NS both to maintain high meat quality and eradicate the *RYR1* g.1843T allele.

## Simple Summary

Nero Siciliano is a native pig breed mainly reared in an open-air system in the mountainous area of north-eastern Sicily. Despite its economic importance and the high-quality meat products, the breed is currently endangered. As part of a broader project to characterize and support the conservation of this breed, the genetic variability of four candidate genes involved in back fat deposition and growth rate was evaluated to provide useful information for the promotion of Nero Siciliano meat products. By analyzing a representative sample of pigs reared in the original breeding area, high frequencies of gene polymorphisms linked to different growth rates and fat deposition were detected. A deleterious *RYR1* g.1843T allele to cause malignant hyperthermia and pale, soft, exudative meat was detected in seven heterozygous pigs. These results highlight the importance of regular genetic monitoring to promote beneficial traits and eradicate harmful variants, preserving local producers and this endangered breed.

## 1. Introduction

The Nero Siciliano (NS) is an ancient native black pig breed that originated in Sicily and is mainly reared in the mountainous Nebrodi area in the north-east of the island. Pig breeding in Sicily dates back to the Greek and Carthaginian periods, and meat products derived from the black pigs of Sicily (the ancestors of the current NS breed) were already known in Rome by the second century BC [1].

Nowadays, most NS pigs are raised “en plein air” in the Nebrodi Nature Park, in beech and oak forests enclosed by fences. These hardy pigs are well adapted to harsh environmental conditions and are valued for their good reproductive performance, disease resistance and meat production. They therefore represent an important resource for the local economy [2,3]. The high quality of NS meat products results from several factors, including genetics, diet and nutrition, environment, and traditional techniques that give cured meats their unique flavor and taste. By exploiting marginal areas and feeding on natural food resources rich in aromatic and antioxidant compounds, as well as monounsaturated fatty acids, the pigs grow slowly but produce tasty meat and fat [4,5], which is used to produce high-quality fresh meat that is mainly transformed into cured hams and other cured meats.

Due to the high added value recognized to its products, a genetic authentication and traceability protocol using a set of SNP markers has been developed for the economic enhancement of Nero Siciliano-only products [6]. Despite its economic and ecological importance and its well-appreciated meat products, the NS is endangered and is officially included in the national conservation programme managed by the Italian Pig Breeders Association (ANAS). Therefore, the FAO DAD-IS system classified the breed as “at risk”, with a local risk status of “endangered maintained”. Currently, the population numbers 4937 pigs, and the census shows a decreasing trend, with 136 boars and 505 breeding sows (last updated on 7 October 2024).

Genetic information on Italian autochthonous pig breeds is still limited. In these local breeds, which are poorly genetically managed and not included in selection and genetic improvement programs, investigating polymorphisms at relevant candidate loci associated with productive traits may provide helpful insights, especially for characterizing breeding stock employed in natural on-farm mating. Taking structural and scientific action to enhance knowledge of genetic variability is essential to promote production performance and genetic improvement of the NS pig breed, to preserve this valuable resource and meet the challenges of the market, climate change and production sustainability. Research activities carried out under the REDSUS project aimed at the “Improvement of the profitability of the Nebrodi Nero Siciliano Pig breeding. Actions on animals and products in respect of animal welfare and environmental sustainability” have focused on the genetic characterization of breeding stock for loci known to influence meat production and quality. Genetic characterization of livestock populations is essential for developing strategies and techniques that protect local breeds at risk and enable more defined and effective selection programs. Furthermore, analyzing intra-breed variability is essential for preserving this genetic resource, its breeding system, and traditional products [7,8].

In the “en plein air” breeding system, the evaluation of growth potential is limited due to restricted information regarding feed intake and nutritional value of feed. Although the productive performance of NS has been little investigated and only a few studies have collected data on meat production in an extensive system [9,10], it is well known that the average daily weight gain and fat deposition in NS are, respectively, much lower and much higher than in modern, selected breeds [11]. These traits can be genetically enhanced. Some candidate loci have been identified in Sus scrofa for meat production and body composition; however, these genes have been scarcely investigated in the NS breed.

The aim of this study is to evaluate the genetic variability of some candidate genes involved in feed intake, growth rate, fattening and in back fat deposition—Melanocortin-4 Receptor (*MC4R*), Ryanodine Receptor 1 (*RYR1*)-Alothane, Class 3 Phosphoinositide 3-Kinase (*PIK3C3*), and Leptin (*LEP*)—in order to provide useful information for maintaining and enhancing the quality and production of NS pork as a valuable tool for its conservation and economic self-sustainability.

## 2. Materials and Methods

### 2.1. Animal Sampling and DNA Extraction

A representative sample of 87 NS pigs (18 boars and 69 sows) was collected from 9 farms (8–14 pigs per farm) in the Nebrodi area, the breed’s original and most traditional breeding area. Only minimally related animals were selected from each farm, taking into account the known genealogical records. The sex ratio reflects that determined in the whole NS population. Peripheral blood was collected in EDTA vacutainer tubes by authorized veterinarians as part of the National Animal Health Service’s routine sanitary screening, in accordance with general recommendations for animal welfare. All efforts were made to minimize animal suffering in line with the European Council’s recommendations on animal care (Directive 2010/63/EU). Genomic DNA was extracted from blood using DNeasy Blood and Tissue Kits for DNA isolation (Qiagen, MD, USA). The quality and concentration of the DNA were quantified using a NanoDrop 1000 spectrophotometer (NanoDrop Technologies, Wilmington, DE, USA).

### 2.2. Genetic Characterization

Nine loci in four candidate genes (*MC4R*, *RYR1-Alothane*, *LEP*, *PIK3C3*) were genotyped using different PCR-RFLP methods and DNA sequencing. Primers were synthesized from Eurofins Genomics (Ebersberg, Germany). The list of analyzed genes and loci, the methods, and the references are reported in Table 1. Genotypes at *MC4R c.-135 C>T* and *c.707 G>A* were analyzed by direct Sanger sequencing. The remaining seven loci were characterized by the PCR-RFLP method, and in order to confirm the digestion results, a subset of six to nine samples with different genotypes for each locus was sequenced. The target DNA sequence for each locus was amplified starting from 50 ng/µL of genomic DNA in a total reaction volume of 50 µL using the MiniAmp Plus thermal cycler (Applied Biosystems, Foster City, CA, USA). The PCR products were purified using Exo-SAP digestion and then dideoxy sequencing was performed using the BigDye™ Terminator v3.1 Cycle Sequencing Kit (Thermo Fisher Scientific Inc., Waltham, MA, USA). Sequencing reactions were performed bidirectionally on an Applied Biosystems 3130 genetic analyzer (Applied Biosystem, Foster City, CA, USA). Chromatograms were visualized using BioEdit software version 7.2.5 (Tom Hall, Ibis Biosciences, Carlsbad, CA, USA), then aligned and compared with the respective reference sequences (GenBank Acc. Num. *FJ357500.1*, *M91451.1*, *AF026976*, *U66254.1*, *AY823302.1*) using MEGA X v.11 [12].

### 2.3. Statistical Analysis

Departure from Hardy–Weinberg genetic equilibrium was evaluated using the χ^2^ test [13]. The software PHASE ver. 2.1, which implements a Bayesian statistical method, was used to reconstruct haplotypes at the *MC4R* and *LEP* loci from allelic frequencies within each gene in each population [14]. The threshold frequency for determining haplotypes was set to 1%. The main descriptive population statistics (Ho, He, Fis and Shannon index) were estimated using “GenAlEx 6.51b2” software version 6.5 [13].

**Table 1:** List of the genes and polymorphic sites analyzed.

Gene	Acc. Num.	Site	SNP	AminoAcids	Primers Sequence (5′-3′)	Amplicon (bp)	Method	Ref
***MC4R***	FJ357500.1	5′ UTR	−780 C>G	-	GTGGCGAAGGTCACAATGG AGTGGCTCCTCCTCTGCTT	640	PCR-RFLP	[15]
			c.135 C>T	-	TCTTCTCCCAATAGCACAGC GGAAACGCTCACCAGCATA	536	Sequencing	[15]
		Ex 1	c.175 C>T rs81221060	p.Leu59Leu	CAGGTCAGAGGGGATCTCAA GTGCAGACTGCCCAGATACA	568	PCR-RFLP	[15]
			c.707G>A rs81221061	p.Arg236His	TCGATTGCAGTGGACAGGTA GAAAATGCTGTTGTGAAGCA	663	Sequencing	[15]
			c.1426G>A rs81219178	p.Asp298Asn			PCR-RFLP	[15]
***RYR1***	M91451.1	Ex 17	c.1843C>T rs706913914	p.Arg615Cys	GTGCTGGATGTCCTGTGTTCCCT CTGGTGACATAGTTGATGAGGTTTG	134	PCR-RFLP	[16]
***LEP***	AF026976	3′ UTR	g.2728G>A rs3475337814	-	CCCTGCTTGCACTTGGTAGC CTGCCACACGAGTCTTGCTC	658	PCR-RFLP	[17]
	U66254.1	Ex 2	c.3469 T>C rs45431504	p.Leu72Leu	AACAGAGGGTCACCGGTTTG TTTGGAAGAGCAGCTTAGCG	486	PCR-RFLP	[18]
***PIK3C3***	AY823302.1	Ex 24	c.2604 C>T rs81211045	p.His826His	ATTTCGTCTAGACCTGTCCG TGAATCTGTTCTACCACCGC	102	PCR-RFLP	[19]

## 3. Results and Discussion

All the analyzed loci were polymorphic in our sample, except for one (*MC4R* c.135 C>T). The results of genetic characterization are presented in Table 2 and Table 3 for the entire population and the subpopulations of boars and sows.

The polymorphisms here analyzed by PCR-RFLP (Table 1) were confirmed by sequencing 6–9 samples with different genotypes. The sequences did not reveal any new mutations.

### 3.1. Genetic Variation at MC4R Gene

The *MC4R* gene encodes the G-protein-coupled melanocortin-4 receptor, which plays an important role in controlling feed intake, energy balance, and fatness in mammals by binding to its ligands, such as the α-melanocyte-stimulating hormone (α-MSH) and the agouti-related protein (AgRP) [20,21]. The receptor is mainly expressed in cells of the central nervous system and has been reported to act as a pivotal mediator of leptin, being involved in the leptin feedback control of food intake, body weight, and energy homeostasis [19]. In pigs, the *MC4R* gene has been mapped on chromosome SSC1 q22-q27 [22], a region encompassing the largest number of economically important QTL in pigs [23,24]. The *MC4R* locus has therefore been reported to be associated with many economic traits, such as backfat, food intake and daily weight gain. The gene consists of a large single exon spanning 999 kb, and several SNPs, including two missense substitutions, have been identified in the coding region.

In this study, five SNPs have been characterized in the *MC4R* gene (Table 1) located in the 5′UTR region and in exon I. Polymorphisms in the 5′UTR region can alter gene expression by changing transcription factor binding sites. Considering this, the −780 C>G locus was investigated in Nero Siciliano pigs. This locus exhibited only two genotypes with the balanced allele frequencies approaching 50%. However, this locus is not in genetic balance within the population, or within the subpopulations of boars and sows, due to a significant excess of heterozygotes (Table 2 and Table S1). In the literature, the −780 C allele has been associated with increased dorsal and lumbar lard thickness and reduced growth rate [15].

A 536 bp fragment of the MC4R gene was sequenced, covering part of the 5′ UTR and part of the coding sequence (*Acc. Num FJ357500.1*). The site described as c.135 C>T by Fan et al. [15], but not annotated as a SNP in the Ensembl dataset, was found to be monomorphic for the T allele in our sample of NS. In a study carried out on Italian Large White pigs, the c.135 C>T SNP occurred at a very low minor-allele frequency and was, therefore, excluded from subsequent association tests [25]. In our sample of 87 NS pigs, direct sequencing revealed four heterozygous variants (in two sows): three of these map to known Ensembl entries—g.1181 T>C (rs81221059), g.1201 T>G (rs330973696), and g.1490 C>T (rs81221060)—and one appears to be novel—g.1311 G>A (Figure S1). Except for the synonymous SNP at position g.1490 in the coding region, all other variants are located in the 5′ UTR region.

The SNP c.175C>T is a synonymous mutation p.Leu59Leu. The polymorphism observed at this locus in NS is quite interesting. The C allele and the CC genotype are predominant in the population, but the locus is not in Hardy–Weinberg equilibrium (Table 2). Furthermore, different allelic and genotypic distributions have been observed in sows and boars (Table 2); in fact, the C allele, identified in the ISU Berkshire×Yorkshire resource population by [15] as potentially associated with faster growth and reduced back-fat thickness, is more frequent in sows than in boars.

Missense mutations can modify the protein’s structure and function. In pigs, the *MC4R* c.707 G>A SNP results in an amino acid substitution in the G-protein sequence at position 236, and this variation is, therefore, commonly known as p.Arg236His. A balanced polymorphism is present in Nero Siciliano at this locus, with the allele G being the major one (0.63) (Table 2). In the literature, the G allele (Arg236) has been associated with major body weight gain and reduced back fat thickness than the A allele [15].

The SNP *MC4R* c.1426 G>A determines a substitution Asp298Asn in the seventh transmembrane domain of the G protein receptor, with the Asp residue well preserved in MCRs and the Asn residue highly conserved in most other G-protein coupled receptors (GPCRs) [22]. As the highly conserved residues play important roles in ligand binding or intracellular signaling [26], this variant may have distinct functional capabilities in the regulation of food intake and body weight, and it is the most studied *MC4R* locus in several pig breeds. Allele A, variant (p. 298Asn), has been suggested to be associated with higher growth rate and lower fatness; however, the effect of this substitution on the function of the receptor is still controversial, limiting its use in practical pig breeding [15].

In NS, this polymorphism is fairly balanced, with the G allele (p.298Asp) and the heterozygous genotype having frequencies close to 50% (0.55 and 0.54, respectively) in the population; however, interestingly, the A allele (298Asn) is the most common in boars (0.61), in contrast to the situation in sows (0.41), suggesting sex-specific selection for faster weight gain in males at this locus. Muñoz et al. [27] found that the A allele showed intermediate frequencies (0.30–0.70) in several other Italian and European local breeds (Apulo-Calabrese, Cinta Senese, Gascon, Krškopolje, and both Lithuanian breeds, Moravka, and Sarda). Recently, Valluzzi et al. [28] reported that allele G and genotype GG were largely predominant in Nero Lucano, another rustic Italian local population of black-coated pigs reared in semi-extensive systems. In the literature, Hirose et al. [29] reported allele A and homozygous genotype AA were the most common in a genetically improved Japanese Duroc population selected for faster growth rate, backfat thickness (BFT) and intramuscular fat content (IMF) in the loin. These results may reflect selective pressure for these productive traits in improved breeds. Indeed, allele A (Asn 298) was found to be associated with increasing daily gain, higher lean meat percentage and reduced backfat thickness across different breeds in Lithuanian White pigs as well as in both Italian Duroc and Large White populations [30,31].

According to Van den Broeke et al. [32], the *MC4R* c.1426G>A locus affected meat production and quality. Pigs with the AA genotype showed a lower dressing percentage, ham width and muscle thickness, but thicker fat, resulting in a lower lean meat percentage compared to GG pigs. In addition, interestingly, AA boars showed significantly higher levels of the boar taint compounds androstenone, skatole, and indole in their fat when compared to GG boars, but overall, these increases did not affect the sensory analysis of the meat and fat samples. Recently, Zhang et al. [33] investigated the functional impact of the substitution p.Asp298Asn, revealing that the Asn298 receptor exhibits reduced basal activity and lower surface expression of MC4R compared to Asp298. This suggests that it could impact the satiety signal and energy balance and serve as a valuable selection marker in pig breeding programs.

### 3.2. Genetic Variation at RYR1, LEP and PIK3C3 Genes

The Ryanodine Receptor1 (*RYR1*) gene, located on porcine chromosome 6 (SSC6q12) [34], encodes the tetrameric Ca^2+^ release channel of the skeletal muscle sarcoplasmic reticulum. A single-base mutation on the *RYR1* g.1843 C>T gene causes an amino acid substitution (Arg615Cys) resulting in uncontrolled Ca^2+^ release and subsequent muscle hypermetabolism [35]. The mutation is associated with both Malignant Hyperthermia (MH) and the inherited myopathy and meat defect known as PSE (Pale Soft Exudative). Historically, this polymorphism is more common in the Pietrain breed, known for its particularly high lean meat content and efficient feed conversion ratio [36]. The RYR1 g.1843 T allele was introduced into commercial pig breeds through the Pietrain boars, often used in selection programs aiming to reduce back fat and to increase lean yield. The defective allele *RYR1* T alters the membrane properties of the sarcoplasmic-reticulum membrane in skeletal muscle fibers, leading to increased release of calcium ions in response to various stressors [37]. In pigs carrying the defective allele *RYR1* T, Fiedler et al. [38] reported increased mean fiber type diameters and glycolytic metabolic potential, a more rapid post-mortem pH decline, and higher drop loss. The wild-type genotype CC showed a statistically significant effect on the average daily gain and backfat thickness, probably due to greater fatness and lower lean meat content [37].

In NS, the *RYR1* g.1843 C>T locus exhibits very low variability and conforms to Hardy–Weinberg equilibrium. The T allele was identified only in seven heterozygous pigs (six sows and one boar) across three farms, although one farm showed a quite allele frequency (0.29). This allele, which is associated with the PSE, Porcine Stress Syndrome (PSS) and Malignant Hyperthermia (MH) [39], can be a cause of major economic losses. The Italian Pig Breeders Association (ANAS) chose to eradicate it from the Italian breeds, mainly used to produce high-quality branded products, such as “Suino Mediterraneo” and “Gran Suino Padano”, because of the deleterious effects of the mutation on meat quality, particularly in cured products. Local breeds, not crossed with cosmopolitan breeds and especially with the Pietrain, are expected to be free from this mutation, as reported for several Mediterranean local breeds [40,41,42], including Mora Romagnola [27] and Nero Lucano [28]. Other authors reported the presence of the Halothane gene at low frequency in most of the Italian breeds, including Nero Siciliano [16,27] Casertana, Apilo-Calabrese, Sarda [27] and Cinta Senese [27,43]. In contrast, high frequencies were found in some other Mediterranean breeds, probably due to historical crossbreeding with Pietrain and Landrace breeds [42,44]. In NS, the presence of the RYR1 g.1843T allele, which is detrimental to meat quality and animal welfare, poses a threat to the breed and highlights the need for careful control of matings.

The *LEP* gene encodes the protein hormone leptin, an adipocytokine secreted mainly by white adipose tissue. This hormone plays a central role in the control of feeding, energy balance and metabolism [45]. It acts on satiety centers in the hypothalamus, which control feed intake, energy balance, body weight, and metabolism through physiological and endocrine mechanisms. In pigs, the *LEP* gene has been mapped to chromosome SSC18q13-q21 [46,47] and consists of three exons: the first is a short untranslated sequence, while the second and third are the coding sequence [48]. Two SNPs have been characterized in NS: g.2728 G>A, located in 3′UTR region, and c.3469 T>C, a synonymous nucleotide substitution identified in the second exon [18,49].

In the NS population, the locus g.2728 G>A shows all three genotypes and a significant deviation of the genotype frequencies from the expected distribution at equilibrium (Table 2), with allele G and genotype AG being the most frequent. The c.3469 T>C transition showed only two genotypes, in our sample of NS, with the T allele and TT genotypes largely predominant in the population, particularly among boars rather than in sows (Table 3). In the literature, many association analyses showed that this polymorphism is significantly associated with production traits such as average daily weight gain [17,18,50]. Kennes et al. [17] found higher average daily gain (ADG) in TT pigs versus CT heterozygotes; de Oliveira Peixoto et al. [50] confirmed that TT animals had the highest body weights throughout the early growth period in an F_2_ Duroc × Landrace cross, whereas CT heterozygotes higher average daily gain and a more efficient feed conversion during the later fattening period; in contrast Chao et al. [18] reported that the C allele predominates in Western commercial breeds being significantly associated with improved growth performance. This contradictory evidence has limited the use of this marker in practical pig breeding so far.

The phosphatidylinositol 3-kinase catalytic subunit type 3 (*PIK3C3*) gene encodes for a protein, phosphoinositide 3-kinase, class 3, which is essential for mammalian development because of its role in performing many specific cellular functions as well as the metabolic activity of insulin. In addition, the gene is mapped to a QTL region on chromosome 6q22-23 [51], so it appears to be a functional and also a positional candidate gene related to the fat deposition trait in domestic pigs. In the literature, the SNP g.2604 C>T, located in exon 24, has been reported to be associated with various production traits; the C allele increased average daily gain, backfat thickness and intramuscular fat in Duroc pigs [52] and body weight and backfat in Landrace x Korean native crosses [19].

In our sample of NS, this SNP showed two genotypes, a strong predominance of the C allele together with a significant excess of heterozygotes in the population (Table 2). The C/T SNP of porcine *PIK3C3* could be utilized as a possible genetic marker to select swine that possess a low back fat thickness and carcass fat content at the slaughter weight [19]. The locus is not in Hardy–Weinberg equilibrium in the population or in both male and female subpopulations (Table 3). Noteworthy is the different distribution of alleles and genotypes in the two subpopulations, which could be the result of different selective pressures towards higher growth rate and lower fat deposition in boars and sows. Recently, a high level of genetic variability with heterozygosity of 0.5 has been reported in Nero Lucano, another local population in Southern Italy [28].

### 3.3. Haplotypes at MC4R, LEP and Genetic Diversity Parameters in Nero Siciliano

In NS, the *MC4R* gene resulted quite polymorphic with four out of five variable loci. In this representative sample of the breed, a total of 16 haplotypes with frequencies greater than 1% were inferred within the *MC4R* gene (Table S1). Haplotype frequencies ranged from 0.156 to 0.005. The highest frequency was recorded by the CTCGG haplotype (0.156 ± 0.017), followed by CTCGA (0.148 ± 0.017). The first four haplotypes accounted for more than 50% of the total frequency (0.595).

All the possible haplotypes were identified within *LEP* gene but among these, GT (0.472) and AT (0.436), were by far the most frequent ones and together accounted for more than 90% of the total frequency (0.91) in this breed (Table S1). In contrast, Valluzzi et al. [28] reported all four possible haplotypes in partial linkage disequilibrium in the Nero Lucano pig.

The genetic diversity parameters obtained for each segregating marker are shown in Table 4. The values of observed heterozygosity (Ho) and expected heterozygosity (He) per locus ranged from 0.080 to 0.977 and from 0.077 to 0.500, respectively, with overall values of 0.450 ± 0.1250 for Ho and 0.314 ± 0.071 for He. Due to an excess of heterozygotes at some loci, *MC4R* (−780 C>G), *LEP* (g.2728G>A), and *PIK3C3* (c.2604 C>T), Fis values were often negative, ranging from −0.955 to 0.284, with a mean of −0.296 ± 0.142. These values indicate recent migration, crossbreeding or outbreeding events. Accidental hybridisation with wild boars could also explain these values. Indeed, Genualdo et al. [53] have reported hybrids resulting from occasional crosses with wild pigs, which are found in the Nebrodi Mountains, where NS pigs are raised in “en plein air” and extensive systems. A subsequent study, analysing sperm DNA integrity, sperm meiotic segregation, and nuclear spatial organisation in a NS boar–pig hybrid, revealed that the hybrid had a high proportion (64%) of motile spermatozoa with regular chromosome composition and specific spatial distribution [54]. These studies confirmed that boar–pig hybrids pose a threat to the conservation of indigenous pig breeds reared in extensive systems.

The breed genetic diversity parameters observed in NS are higher than those recorded in 20 local European pig breeds [27]. The mean number of alleles (Na = 1.889), the mean number of effective alleles (Ne = 1.582) per locus and the Shannon index (I = 0.460) confirm a good level of genetic diversity at the SNP candidate loci analyzed. These data support the good sampling strategies carried out in the most representative farms of the traditional breeding area of these pigs.

## 4. Conclusions

The Nero Siciliano pig, reared “en plein air” in the Nebrodi mountains, is an endangered breed appreciated for its meat and cured meats. These high-quality food products represent important resources for the exploitation and conservation of this endangered breed. They are the result of several components, including the breed’s genetics. This study provides a detailed analysis of genetic variant distributions at selected candidate genes associated with growth and fattening in a representative sample of Nero Siciliano pigs. Molecular analysis and DNA sequencing revealed a new SNP in MC4R 5′UTR. Furthermore, some alleles, linked to different growth rate and back fat deposition showed high frequencies in the whole sample (*MC4R* c.175C—0.93; *LEP* g.3469T—0.91). Deviations from genetic balance and different distributions in boars and sows were also observed at some loci (*MC4R* −780 C>G; *MC4R* c.175 C>T; *LEP* g.2728 G>A; *PIK3C3* g.2604 C>T). The presence of the *RYR1* g.1843T allele, which is associated with MH and PSE, was detected in three farms and was found at a rather high frequency (0.29) in one of them. Our results confirm substantial genetic variability in the NS breed and suggest the importance of continuous genetic monitoring in order to plan proper breeding strategies. Particular attention should be given to the eradication of the *RYR1* g.1843T allele, which is detrimental to meat quality and animal welfare and causes significant economic losses.

## Figures and Tables

**Table 2:** Genotype distribution, allele frequencies and Hardy–Weinberg equilibrium tests for MC4R polymorphisms in Nero Siciliano pig breed.

Gene	Locus								
*MC4R*	−780 C>G								
	N	Sample	CC	CG	GG	C	G	χ^2^	*p*-Value
	87	Population	2	85	-	51%	49%	79.355	<0.001
	18	Boars	-	18	-	50%	50%	18	<0.001
	69	Sows	2	67	-	51%	49%	61.444	<0.001
	c.135 C>T								
	N	Sample	CC	CT	TT	C	T	χ^2^	*p*-value
	87	Population	-	-	TT	-	100%	-	-
	18	Boars	-	-	TT	-	100%	-	-
	69	Sows	-	-	TT	-	100%	-	-
	c.175 C>T								
	N	Sample	CC	CT	TT	C	T	χ^2^	*p*-value
	87	Population	77	8	2	93%	7%	7.014	0.008
	18	Boars	14	3	1	86%	14%	1.655	0.1982
	69	Sows	63	5	1	95%	5%	4.288	0.0397
	c.707 G>A								
	N	Sample	GG	GA	AA	G	A	χ^2^	*p*-value
	87	Population	33	44	10	63%	37%	0.666	0.414
	18	Boars	6	10	2	61%	39%	0.5130	0.4738
	69	Sows	27	34	8	64%	36%	0.3039	0.5814
	c.1426 G>A								
	N	Sample	GG	GA	AA	G	A	χ^2^	*p*-value
	87	Population	24	47	16	55%	45%	0.699	0.403
	18	Boars	2	10	6	39%	61%	0.5131	0.4738
	69	Sows	22	37	10	59%	41%	0.7740	0.3790

**Table 3:** Genotype distributions, allele frequencies and Hardy–Weinberg equilibrium tests for *RYR1*, *LEP* and *PIK3C3* polymorphisms in the Nero Siciliano pig breed.

Gene	Locus								
*RYR1*	g.1843 C>T								
	N	Sample	CC	CT	TT	C	T	χ^2^	*p*-value
	87	Population	80	7	-	96%	4%	0.152	0.696
	18	Boar	17	1	-	97%	3%	0.015	0.903
	69	Sows	63	6	-	96%	4%	0.142	0.705
*LEP*	g.2728 G>A								
	N	Sex	GG	GA	AA	G	A	χ^2^	*p*-value
	87	Population	11	70	6	53%	47%	32.855	<0.001
	18	Boar	2	13	3	47%	53%	3.627	0.0568
	69	Sows	9	57	3	54%	46%	30.4917	<0.001
	c.3469 T>C								
	N	Sample	TT	TC	CC	T	C	χ^2^	*p*-value
	87	Population	70	16	-	91%	9%	0.904	0.342
	18	Boar	16	2	-	94%	6%	0.0623	0.8029
	68	Sows	54	14	-	90%	10%	0.8955	0.3440
*PIK3C3*	g.2604 C>T								
	N	Sex	CC	CT	TT	C	T	χ^2^	*p*-value
	87	Population	12	75	-	57%	43%	49.931	<0.001
	18	Boar	-	18	-	50%	50%	18	<0.001
	69	Sows	12	57	-	59%	41%	34.1687	<0.001

**Table 4:** Observed (Ho) and expected heterozygosity (He) and fixation index (F) values by locus in Nero Siciliano.

Gene	SNP	Ho	He	F
*MC4R*	−780 C>G	0.977	0.500	−0.955
	c.135 C>T	-	-	-
	c.175 C>T *rs81221060*	0.092	0.128	0.284
	c.707G>A *rs81221061*	0.506	0.465	−0.088
	c.1426 G>A *rs81219178*	0.540	0.496	−0.090
*RYR1*	c.1843C>T *rs706913914*	0.080	0.077	−0.042
*LEP*	g.2728G>A *rs3475337814*	0.862	0.490	−0.758
	c.3469 T>C *rs45431504*	0.186	0.169	−0.103
*PIK3C3*	c.2604 C>T *rs81211045*	0.805	0.498	−0.615

## Data Availability

Data are available from the corresponding author.

## Supplementary Materials

The following supporting information can be downloaded at: https://www.mdpi.com/article/10.3390/ani15142143/s1, Figure S1: Single Nucleotide Polymorphisms observed in the 5’UTR and in Exon I of the *MC4R* gene in Nero Siciliano pigs. The SNP g.1181 T>C (rs81221059), g.1201 T>G (rs330973696), and g.1490 C>T (rs81221060) map to known Ensembl entries, whereas g.1311 G>A appears to be novel; Table S1: Haplotype distribution at the *MC4R* and *LEP* genes in Nero Siciliano pigs.
