# Supplementary material for: Genetic Variability of Loci Affecting Meat Quality and Production in Nero Siciliano Pig Breed
Source: Animals (Basel). 2025 Jul 19;15(14):2143. doi: 10.3390/ani15142143 (PMC12291818; doi:10.3390/ani15142143)
Supplement: Supplementary file 1 [file animals-15-02143-s001.zip › animals-3729710-supplementary.pdf]

Figure S1: Single Nucleotide Polymorphisms observed in the 5′UTR and in Exon I of the MC4R gene in Nero Siciliano pigs. The SNP g.1181 T>C (rs81221059), g.1201 T>G (rs330973696), and g.1490 C>T (rs81221060) map to known Ensembl entries, whereas g.1311 G>A appears to be novel.

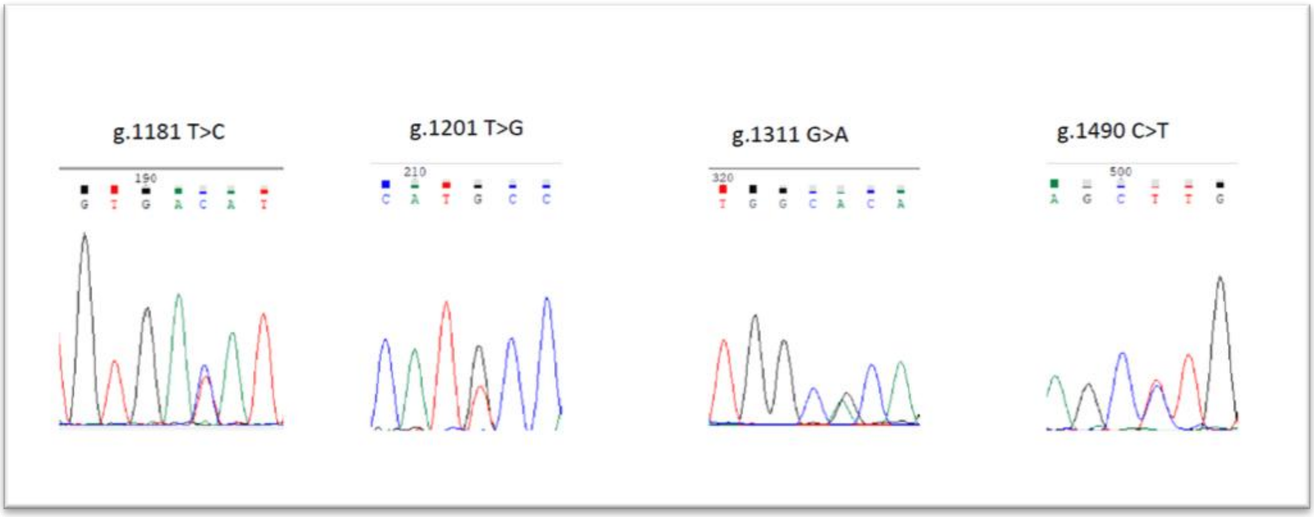

Table S1: Haplotype distribution at the *MC4R* and *LEP* genes in Nero Siciliano pigs

Gene	Haplotype	E (freq)	S.E
MC4R	CTCGG	0.156	0.017
	CTCGA	0.148	0.017
	GTCGG	0.146	0.019
	GTCGA	0.145	0.018
	CTCAG	0.108	0.016
	GTCAG	0.095	0.016
	CTCAA	0.066	0.016
	GTCAA	0.066	0.017
	GTTGG	0.014	0.007
	CTTGG	0.012	0.006
	CTTAA	0.008	0.006
	GTTAA	0.008	0.006
	GTTAG	0.007	0.005
	CTTAG	0.007	0.006
	GTTGA	0.006	0.005
	CTTGA	0.005	0.005
LEP	GT	0,472	0,012
	AT	0,436	0,012
	GC	0,057	0,012
	AC	0,035	0,012
